# Supplementary material for: Architecture of the Tuberous Sclerosis Protein Complex
Source: J Mol Biol. 2021 Jan 22;433(2):166743. doi: 10.1016/j.jmb.2020.166743 (PMC7840889; doi:10.1016/j.jmb.2020.166743)
Supplement: Supplementary data 1 [file mmc1.pdf]

Architecture of the Tuberous Sclerosis Protein Complex

Kailash Ramlaul[1]*, WenchengFu[2]*, Hua Li[2]*, Natàlia de Martin Garrido[1], Lin He[3], Manjari Trivedi[4], Wei Cui[4], Christopher H S Aylett[1†], & Geng Wu[2†]

Supplementary Information

[1] Section for Structural Biology, Department of Medicine, Imperial College London, Exhibition Road, London, SW7 2BB, United Kingdom

[2] State Key Laboratory of Microbial Metabolism, School of Life Sciences & Biotechnology, The Joint International Research Laboratory of Metabolic & Developmental Sciences MOE, Shanghai Jiao Tong University, Shanghai, China

[3]Instrumental Analysis Center, Shanghai Jiao Tong University, Shanghai, China

[4]Institute of Reproductive and Developmental Biology, Faculty of Medicine, Imperial College London, Du Cane Road, London, W12 0NN, United Kingdom

* These authors contributed equally to this study.

† To whom correspondence may be addressed:

C.H.S.A. - c.aylett@imperial.ac.uk

G.W. - geng.wu@sjtu.edu.cn

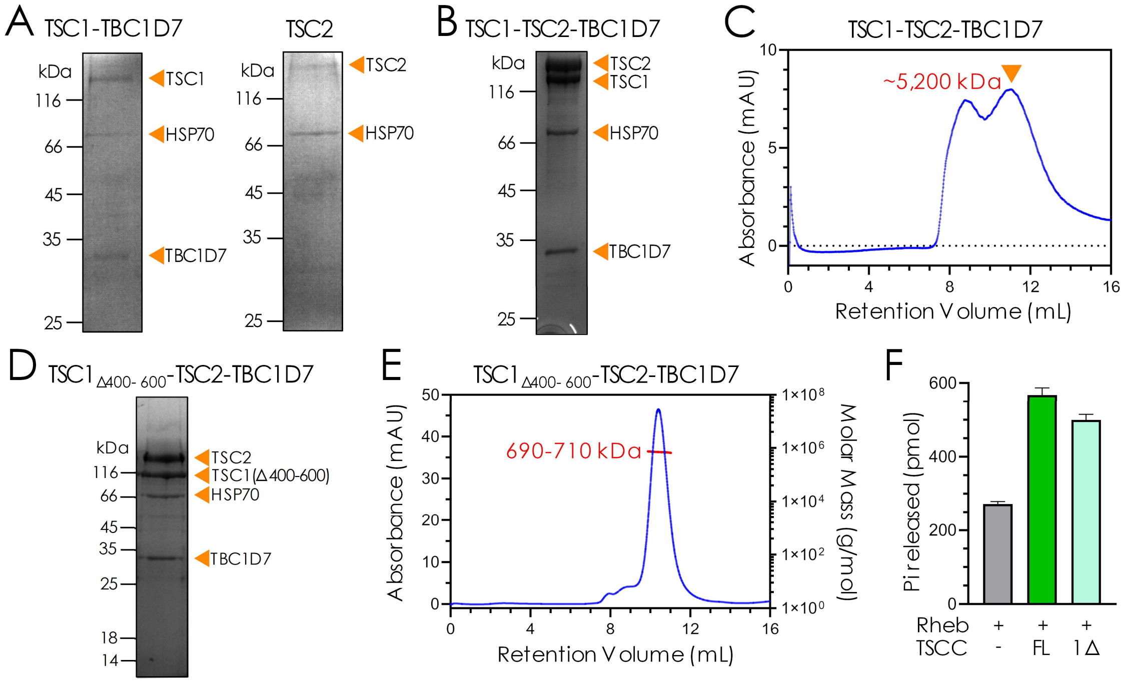

Supplementary Figure 1: Production of functional human holo-TSCC. A. Coomassie-stained SDS-PAGE gels showing TSC1-TBC1D7 alone (left) could be expressed and purified after transient transfection in HEK293F cells, whereas TSC2 alone (right) expressed in much lower quantity and was found to be irreversibly aggregated or degraded. B. Coomassie-stained SDS-PAGE of purified holo-TSCC, demonstrating stoichiometric pulldown of all three components in good yield. C. Size exclusion chromatogram of full-length TSCC. The complex elutes with an approximate molecular weight of 5200 kDa from a Superose 6 size exclusion column, indicating the presence of inter-TSCC interactions leading to higher-order oligomer formation. D. Coomassie-stained SDS-PAGE of purified TSC1(Δ400-600)-TSC2-TBC1D7, indicating that deletion of residues 400-600 did not affect the integrity of the core complex. E. SEC-MALLS chromatogram and molecular weight estimate for TSC1$_{\Delta 400\text{-}600}$-TSC2-TBC1D7. The peak was isotropic, and the molecular weight estimate was well within range of the observed protein components upon later structural examination. F. Results of three repetitions of a malachite green inorganic phosphate release assay conducted with purified Rheb alone, as well as Rheb mixed with full-length TSCC (FL) and TSC1(Δ400-600)-TSC2-TBC1D7 (1Δ), showing a clear increase in GTPase activity in the presence of the TSCC in either full-length or truncated form.

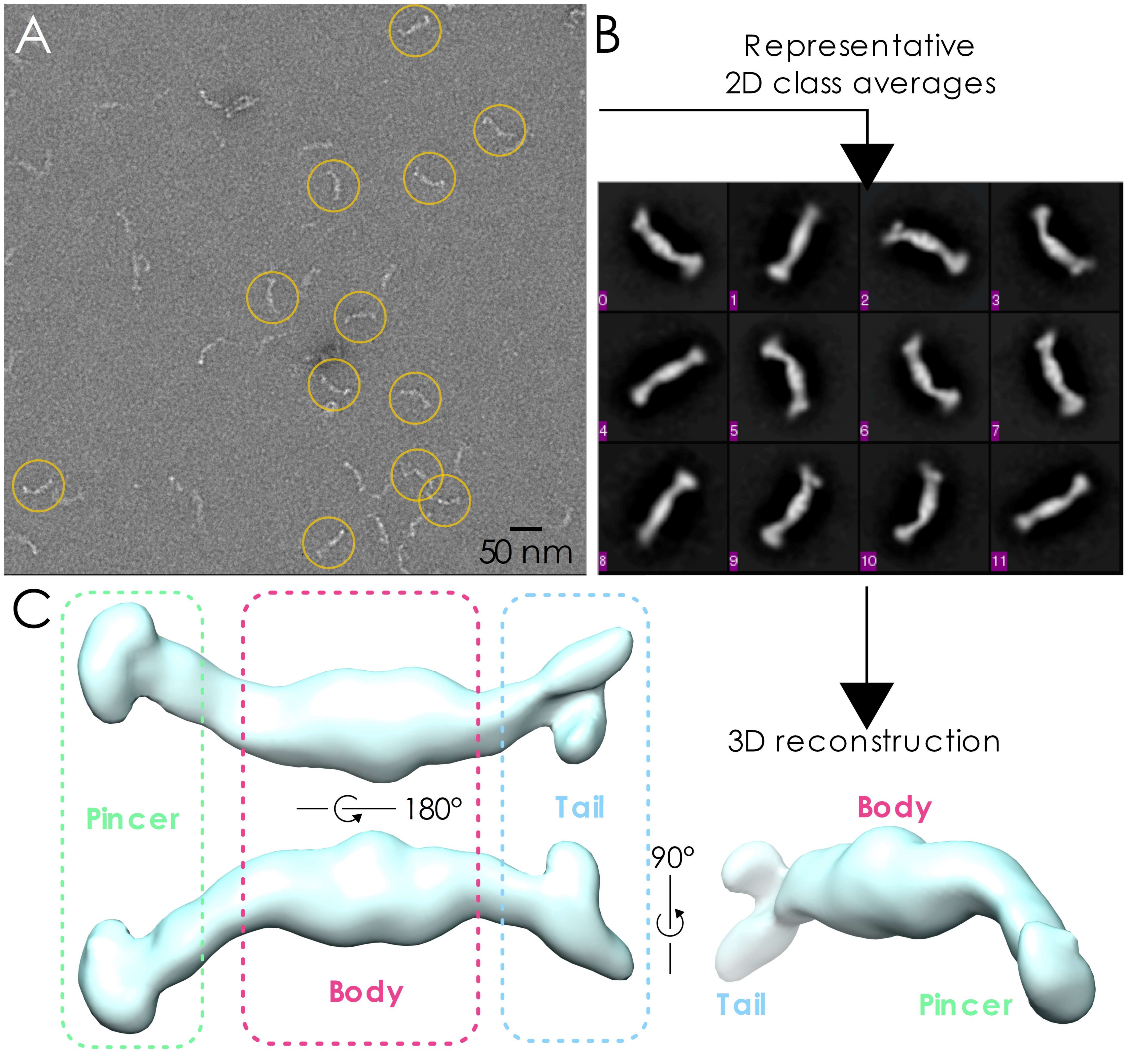

Supplementary Figure 2: Negatively stained EM data of $TSCC_{FL}$. A. Representative negatively stained electron micrograph, clearly showing the elongated structure of the TSCC. Examples of particles are circled in orange. B. Representative 2D class averages from this dataset. C. 3D reconstruction of $TSCC_{FL}$, clearly identifying a tripartite architecture of the full complex. The pincer, body and tail regions could be distinguised based on their low-resolution features.

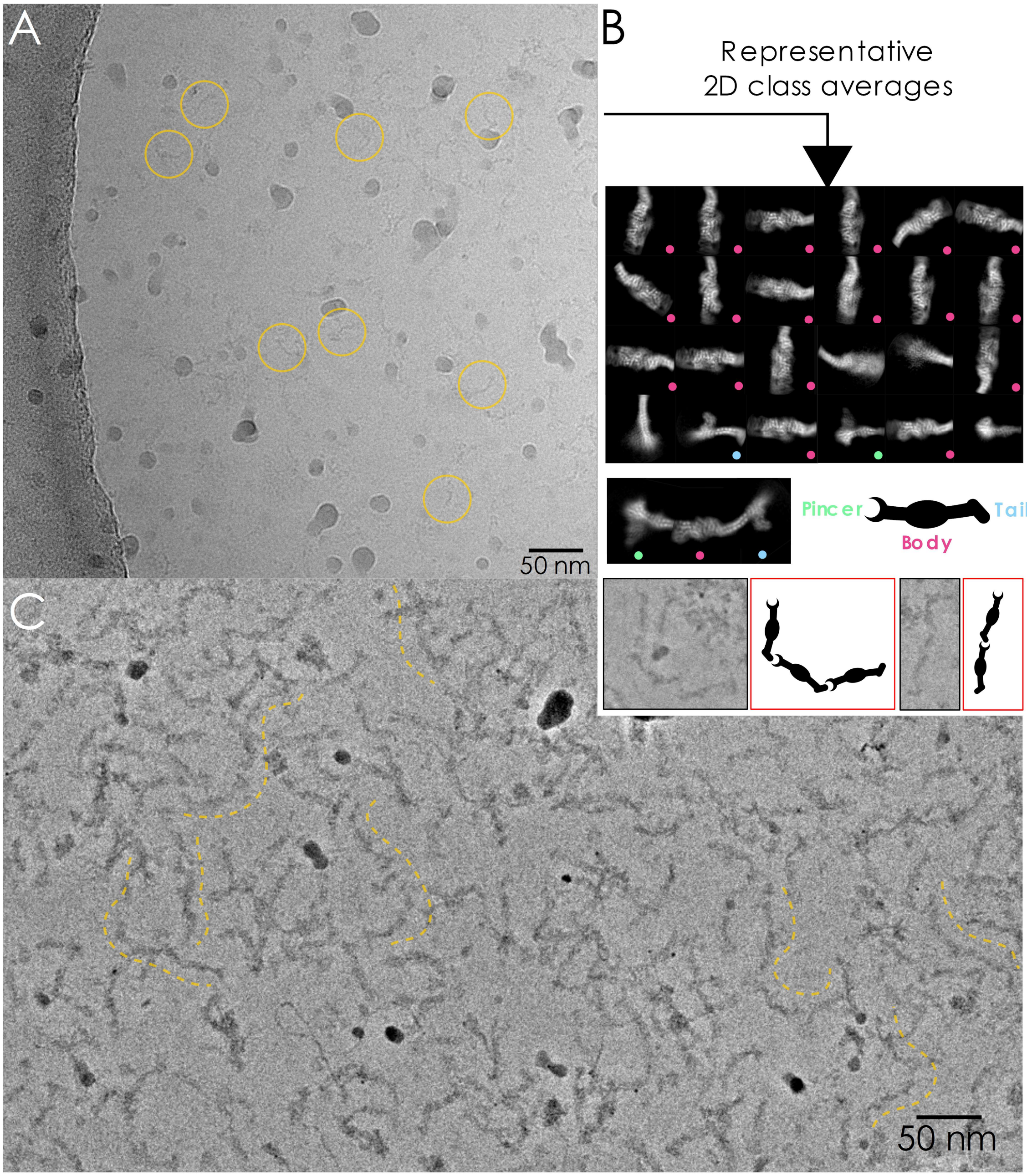

Supplementary Figure 3: Representative micrographs and identification of web-like networks formed by TSCC on grids. A. Representative cryo-electron micrograph of the $TSCC_{FL}$ dataset. B. Representative 2D class averages of $TSCC_{FL}$. Body, pincer and tail views are highlighted by coloured dots corresponding to the key shown below. C. View of cryo-electron micrograph of $TSCC_{FL}$ collected with a Volta phase plate. The increased contrast made it possible to identify TSCC forming higher-order oligomers on the grid (orange dotted lines). (Inset, top right) Close comparison of TSCC oligomer views with our proposed architectural model of TSCC reveals that the complex forms these filaments with a defined, "pincer-to-tail", topology.

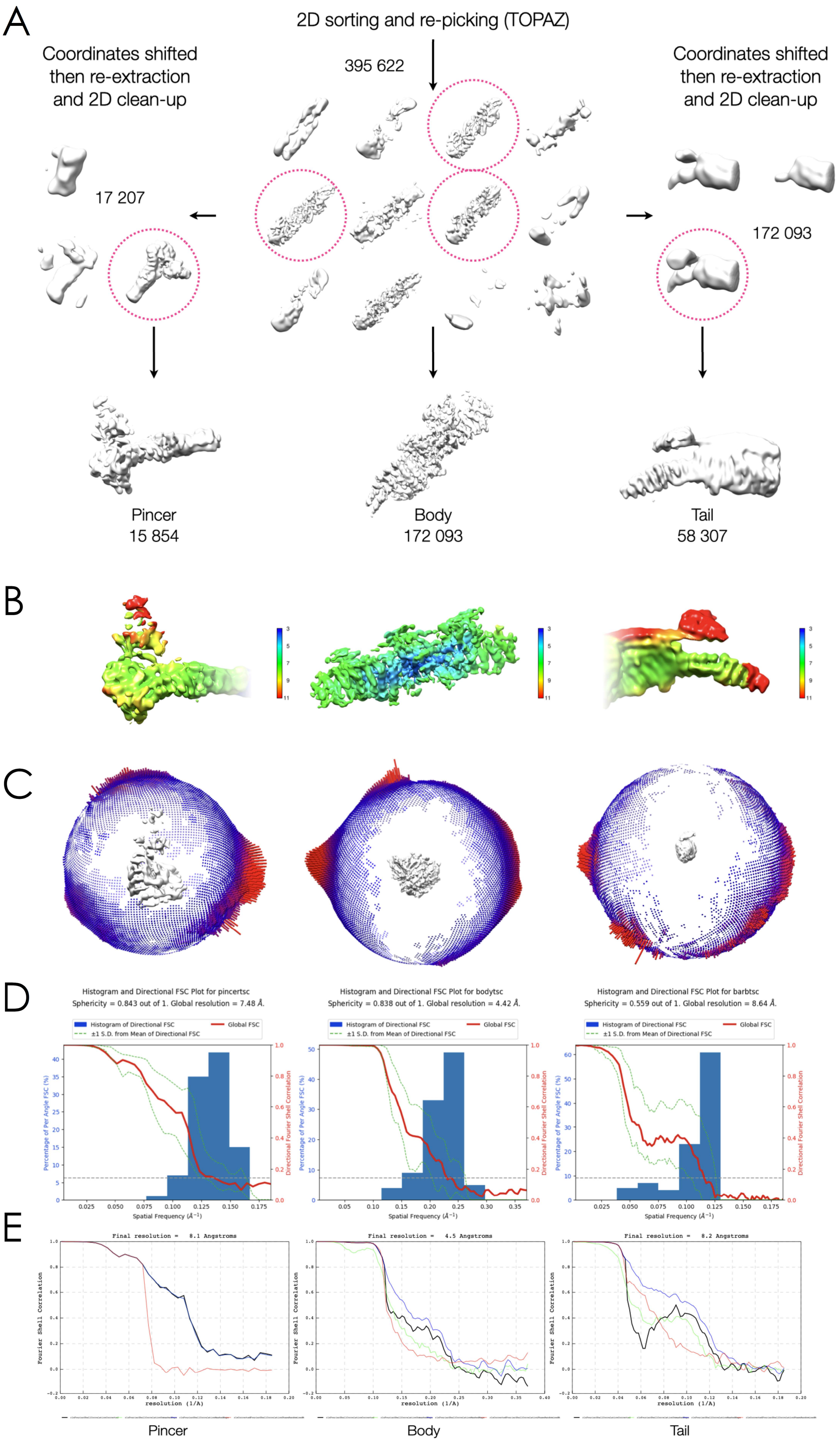
A
2D sorting and re-picking (TOPAZ)
395 622
Coordinates shifted then re-extraction and 2D clean-up
17 207
Coordinates shifted then re-extraction and 2D clean-up
172 093
Pincer
15 854
Body
172 093
Tail
58 307
B
C
D
Histogram and Directional FSC Plot for pincertsc
Sphericity = 0.843 out of 1. Global resolution = 7.48 Å.
Histogram and Directional FSC Plot for bodytsc
Sphericity = 0.838 out of 1. Global resolution = 4.42 Å.
Histogram and Directional FSC Plot for barbtsc
Sphericity = 0.559 out of 1. Global resolution = 8.64 Å.
Histogram of Directional FSC
Global FSC
±1 S.D. from Mean of Directional FSC
Percentage of Per Angle FSC (%)
Directional Fourier Shell Correlation
Spatial Frequency (Å⁻¹)
E
Final resolution = 8.1 Angstroms
Final resolution = 4.5 Angstroms
Final resolution = 8.2 Angstroms
Fourier Shell Correlation
resolution (1/A)
Pincer
Body
Tail

Supplementary Figure 4: Cryo-EM data processing scheme. A. Flow-chart showing data processing procedure (see Materials and Methods for details). Particle numbers are indicated next to each processing step. Each of the “pincer”, “body”, and “tail” regions were processed independently after isolating 2D classes representing each of these views. B. Local resolution maps estimated for each reconstruction using BLOCRES [1]. C. Angular distribution of TSCC particles on the grid after auto-refinement in RELION, demonstrating preferred orientation and absence of end-on views in each case. D. Histogram of Euler angle distribution and directional FSC of each reconstruction calculated using the 3DFSC server [2]. E. The global resolution of each final map by FSC using a cutoff of 0.143 in RELION.

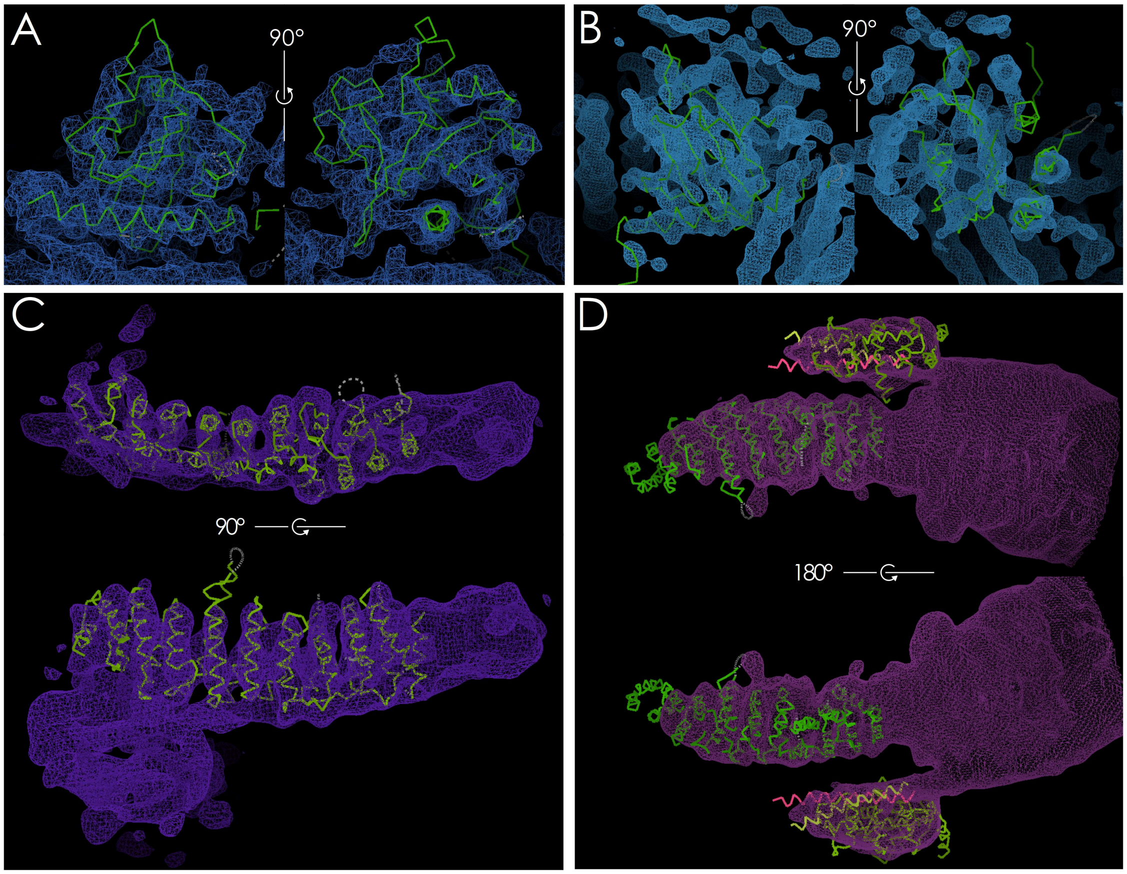

Supplementary Figure 5: Alignment of known crystal structures in cryo-EM reconstructions. A. The *C. thermophilum* TSC2$_{GAP}$ crystal structure (PDB ID: **6SSH**; shown as backbone in green) [3] fits unambiguously into the region of density bound against the TSC2 HEAT repeat within the "body". The human GAP domain is therefore highly structurally homologous. B. The dimerisation domain of the human RapGAP crystal structure (PDB ID: **3BRW**; shown as backbone in green) [4] fits into the dimerisation interface between the two TSC2 HEAT repeats. The topology of the β-sheets is conserved. C. The *C. thermophilum* TSC2 N-terminal HEAT repeat fragment crystal structure (PDB ID: **5HIU**; shown as backbone in yellow-green) [5] fits into the "pincer", indicating the α-solenoid extends from C-terminus, at the dimerisation interface, to N-terminus, at either end of the elongated complex. D. The *C. thermophilum* TSC2 N-terminal α-solenoid, as shown in C. but coloured green, and the TBC1D7-TSC1 crystal structure (PDB ID: **5EJC**; TBC1D7 shown as backbone in yellow-green and TSC1 coiled-coil helices shown as backbone in yellow and pink) [6] fit into density of the "tail" and "barb". The TBC1D7 density is weak, indicating a less well-ordered interaction between the TSC1 coiled-coil-TBC1D7 junction and TSC2, but a track for the TSC1 coiled-coil is clearly visible when compared to the "pincer".

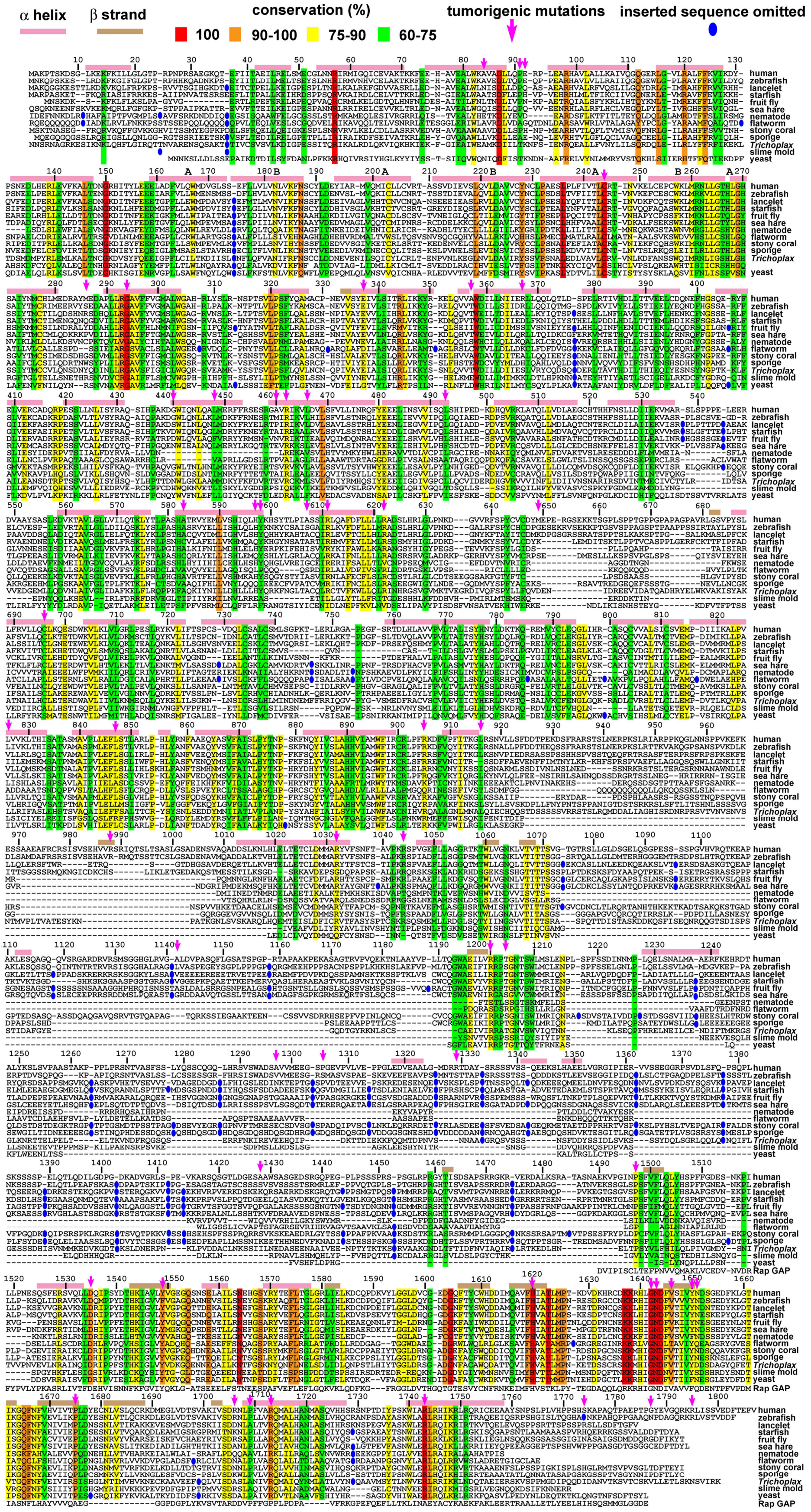
α helix
β strand
conservation (%)
100
90-100
75-90
60-75
tumorigenic mutations
inserted sequence omitted
human
zebrafish
lancelet
starfish
fruit fly
sea hare
nematode
flatworm
stony coral
sponge
Trichoplax
slime mold
yeast
Rap GAP

Supplementary Figure 6: Multiple sequence alignment and secondary structural prediction of TSC2. Human TSC2 displays average sequence conservation with close homologs for most of the protein except the GAP domain, which is highly conserved. Human TSC2 protein (1807 residues) was aligned with orthologues from zebrafish (*Danio rerio*, accession: **XP_021331278**), lancelet (*Bronchiostoma belcheri*, subphylum of Cephalochordata, accession: **XP_019643564**), starfish (*Acanthaster planci*, phylum of Echinodermata, accession: **XP_022103490**), fruit fly (*Drosophila melanogaster*, phylum of Arthropoda, accession: **NP_524177**), sea hare (*Aplysia californica*, phylum of Mollusca, accession: **XP_012937341**), nematode (*Brugia malayi*, phylum of Nematoda, accession: **XP_001893474**), flatworm (*Macrostomum lignano*, phylum of Platyhelminthus, accession: **PAA85829**), stony coral (*Orbicella faveolata*, phylum of Cnidaria, accession: **XP_020601823**), sponge (*Amphimedon queeslandica*, phylum of Porifera, accession: **XP_019853372**), *Trichoplax adhaerens* (phylum of Placozoa, accession: **XP_002116811**), slime mold (*Planoprotostelium fungivorum*, kingdom of Protista, accession: **PRP73953**), and yeast (*Schizosaccharomyces pombe*, kingdom of Fungi, accession: **CAB52735**), as well as human RapGAP by the Clustal W method using MegAlign in the LASERGENE software. The tumorigenic mutations of TSC2 are indicated with magenta arrows.

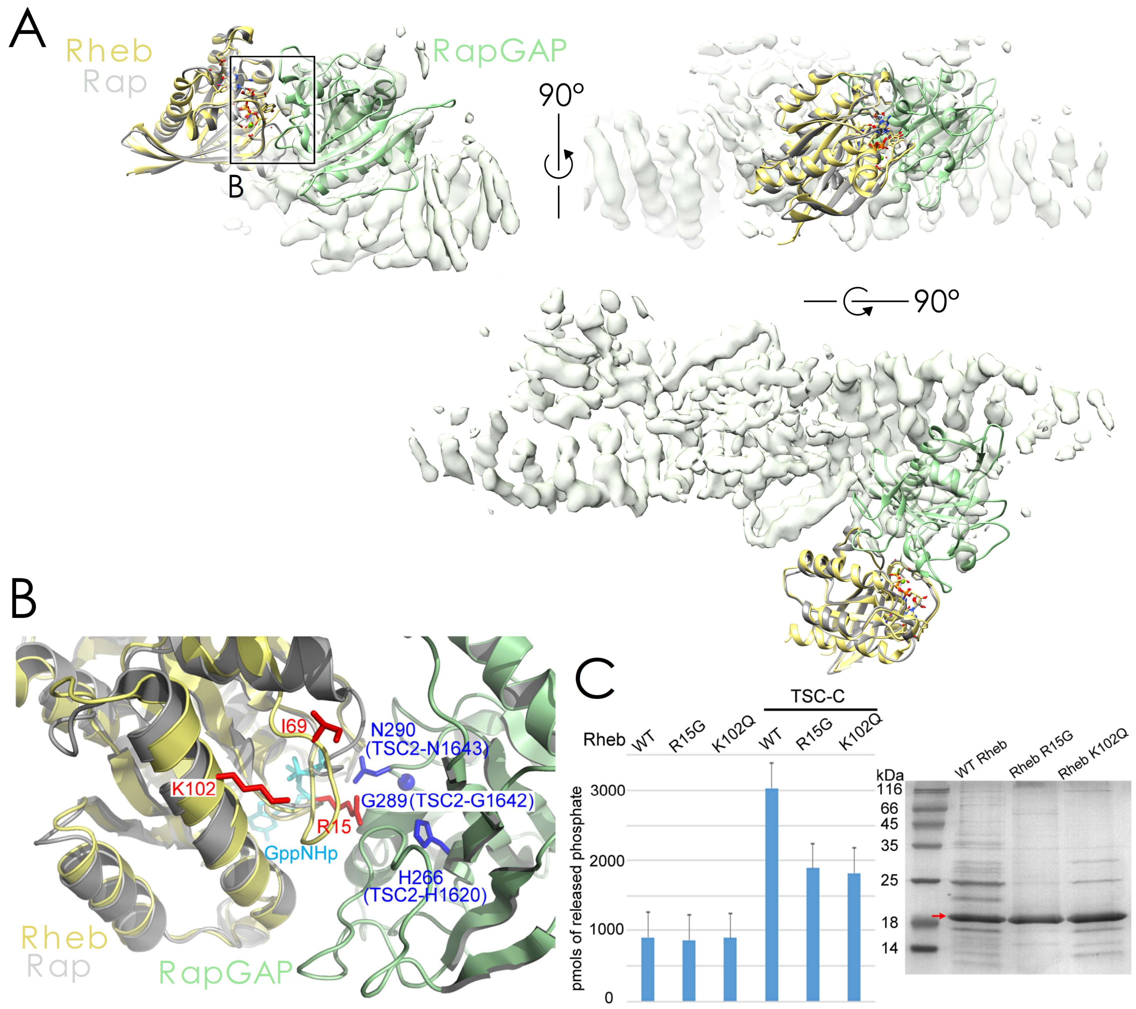

Supplementary Figure 7: Rheb docking and TSC2-GAP-Rheb interactions based on sequence and structural homology to Rap GTPase-RapGAP. A. The crystal structure of Rheb·GTP [7] (PDB ID: **1XTS**) is superimposed onto the crystal structure of the Rap GTPase-RapGAP crystal structure [4] (PDB ID: **3BRW**). The RapGAP domain fitted into density we have assigned to the TSC2 GAP domain in our "body" reconstruction, highlighting how Rheb is captured in this conformation. Rheb is coloured yellow, the Rap GTPase grey, and the RapGAP domain light green. B. The crystal structure of Rheb is superimposed onto the crystal structure of the RapGAP-Rap complex as in A. Rap-contacting residues of RapGAP which correspond to tumorigenic mutations on TSC2 are highlighted in blue. The Rheb residues which are conserved in Rheb homologues but are different in all other Ras family GTPase members are highlighted in red. Interestingly, these residues of Rheb all point toward TSC2-GAP. C. Results of three repetitions of a malachite green inorganic phosphate release assay (left) conducted with purified WT Rheb and two Rheb mutants, R15G and K102Q (right; residues highlighted red as in B), in the absence and presence of TSCC. The assay shows a clear deficiency of

TSCC-stimulated GAP activity by the Rheb mutants relative to WT Rheb, indicating likely involvement of these residues in interaction with TSC2-GAP.

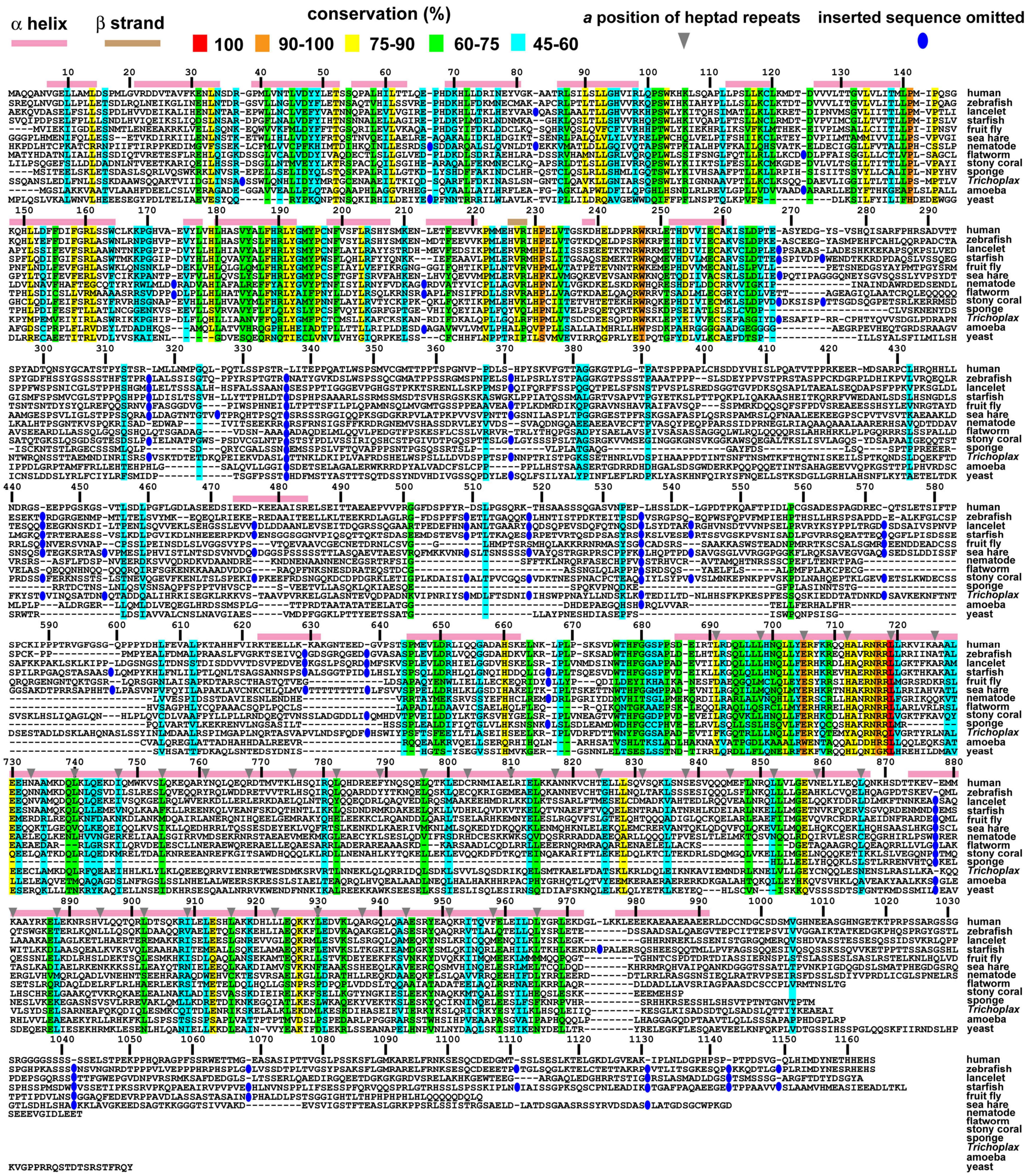

Supplementary Figure 8: Multiple sequence alignment and secondary structural prediction of TSC1. Human TSC1 protein (1164 residues) was aligned with orthologues from zebrafish (*Danio rerio*, accession: **NP_001269321**), lancelet (*Bronchiostoma belcheri*, accession: **XP_019640975**), starfish (*Acanthaster planci*, accession: **XP_022085127**), fruit fly (*Drosophila melanogaster*, accession: **CCB63097**), sea hare (*Aplysia californica*, accession: **XP_005108980**), nematode (*Ancyclostoma ceylanicum*, accession: **EPB75620**), flatworm (*Macrostomum lignano*, accession: **PAA78603**), stony coral (*Stylophora pistillata*, accession: **XP_022782800**), sponge (*Amphimedon queenslandica*, accession: **XP_019858430**), *Trichoplax adhaerens* (accession: **XP_002109434**), amoeba

(*Acanthamoeba castellanii*, accession: **XP_004339658**), and yeast (*Schizosaccharomyces pombe*, accession: **CAA91078**), by the Clustal W method using MegAlign in the LASERGENE software.

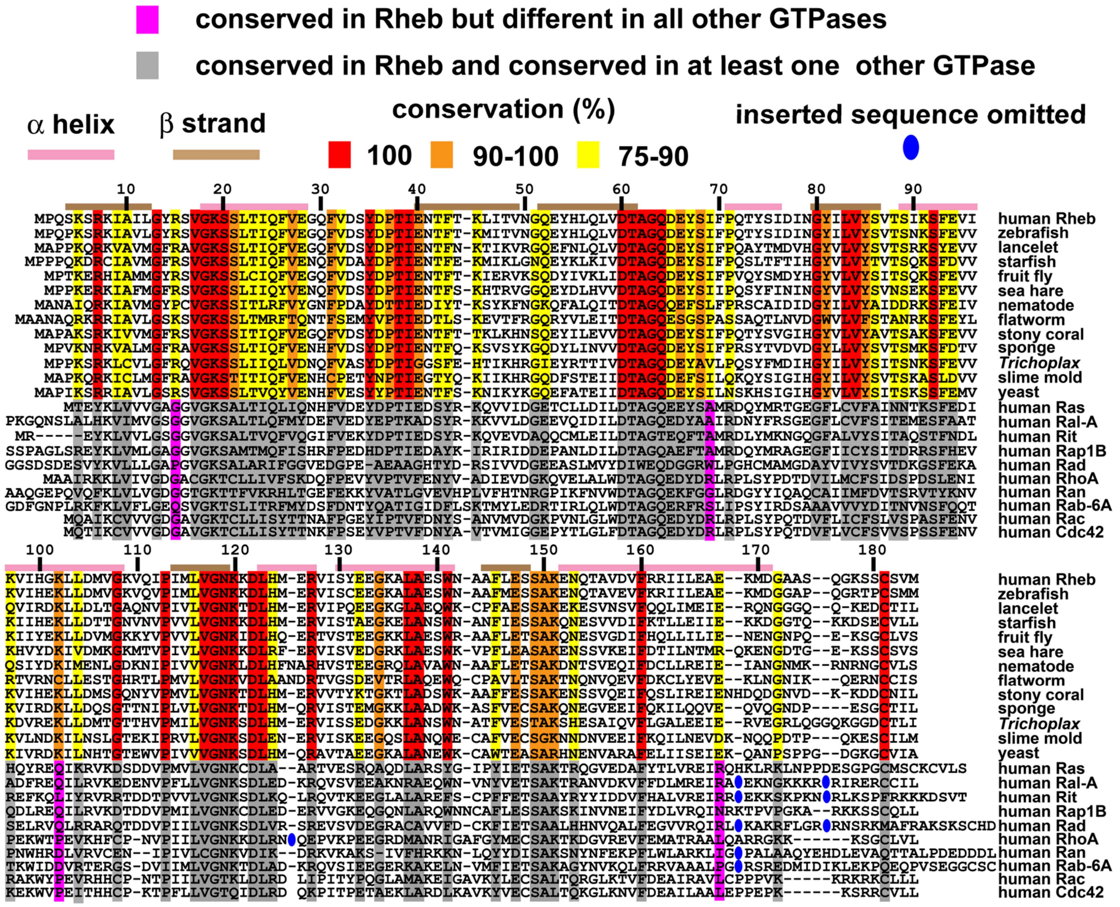

Supplementary Figure 9: Multiple sequence alignment of Rheb and other Ras family members show that R15, I69, and K102 of Rheb are unique among Ras family. Residues unique to Rheb, *i.e.*, those which are conserved in Rheb homologues but are different in all other Ras family GTPases are highlighted in magenta.

Supplementary Table 1. Frequent tumourigenic missense mutations of TSC2 in the tuberous sclerosis database. Tabulated tumourigenic missense mutations of TSC2 with over five entries in the Leiden Open Variation Database:

[http://chromium.lovd.nl/LOVD2/TSC/home.php?select_db=TSC2].

residue	Tumorigenic missense mutations of TSC2				Total entries
A84	A84V (5 entries)				5
P91	P91L (6 entries)				6
E92	E92V (8 entries)				8
C244	C244R (2 entries)	C244Y (3 entries)			5
M286	M286V (10 entries)	M286T (1 entry)			11
G294	G294R (1 entry)	G294E (2 entries)	G294V (2 entries)		5
E337	E337K (5 entries)				5
A357	A357V (5 entries)				5
R367	R367Q (17 entries)	R367P (1 entry)			18
G440	G440S (7 entries)				5
L448	L448P (4 entries)	L448R (1 entry)			5
A460	A460T (11 entries)				11
R462	R462G (2 entries)	R462C (1 entry)	R462H (6 entries)	R462P (2 entries)	11
L466	L466P (4 entries)	L466R (1 entry)			5
L493	L493V (4 entries)	L493P (2 entries)			6
A583	A583T (8 entries)				8
H597	H597Y (3 entries)	H597P (1 entry)	H597R (7 entries)	H597L (1 entry)	12
Y598	Y598H (5 entries)	Y598L (2 entries)			7
A607	A607T (7 entries)	A607S (1 entry)	A607E (1 entry)		9
R611	R611W (44 entries)	R611G (4 entries)	R611Q (87 entries)	R611P (1 entry)	136

R622	R622W (10 entries)	R622Q (1 entry)	R622P (4 entries)		15
M649	M649V (1 entry)	M649L (1 entry)	M649T (6 entries)		8
C696	C696R (4 entries)	C696Y (2 entries)			6
L826	L826M (8 entries)	L826P (3 entries)			11
L847	L847P (5 entries)	L847R (1 entry)			6
R905	R905W (49 entries)	R905G (2 entries)	R905Q (17 entries)		68
L916	L916P (3 entries)	L916R (2 entries)			5
R988	R988C (1 entry)	R988H (1 entry)	R988P (3 entries)		5
R1032	R1032P (8 entries)				8
R1044	R1044K (2 entries)	R1044T (1 entry)	R1044M (2 entries)		5
A1141	A1141T (1 entry)	A1141V (11 entries)			12
R1200	R1200W (28 entries)	R1200Q (1 entry)	R1200P (3 entries)		32
T1203	T1203P (4 entries)	T1203K (1 entry)			5
A1297	A1297T (8 entries)				8
P1305	P1305S (1 entry)	P1305L (5 entries)			6
R1329	R1329H (12 entries)				12
A1429	A1429S (10 entries)				10
P1497	P1497T (3 entries)	P1497S (2 entries)	P1497R (3 entries)	P1497L (4 entries)	12
D1535	D1535Y (2 entries)	D1535A (2 entries)	D1535V (1 entry)		5
Y1549	Y1549N (1 entry)	Y1549H (1 entry)	Y1549C (4 entries)		6
H1620	H1620Y (5 entries)	H1620R (1 entry)			6

G1642	G1642D (5 entries)					5
N1643	N1643H (3 entries)	N1643S (2 entries)	N1643I (1 entry)	N1643K (1 entry)		7
V1646	V1646M (3 entries)	V1646L (2 entries)	V1646G (1 entry)			6
Y1650	Y1650N (2 entries)	Y1650H (2 entries)	Y1650S (1 entry)	Y1650C (4 entries)		9
N1651	N1651H (2 entries)	N1651S (14 entries)				16
P1675	P1675L (55 entries)	P1675Q (2 entries)	P1675R (4 entries)			61
R1706	R1706C (3 entries)	R1706H (2 entries)				5
P1709	P1709L (12 entries)	P1709R (2 entries)				14
R1713	R1713H (9 entries)	R1713P (3 entries)				12
R1743	R1743G (2 entries)	R1743W (47 entries)	R1743Q (49 entries)	R1743P (6 entries)	R1743L (2 entries)	106
S1774	S1774T (9 entries)					9
G1787	G1787S (6 entries)					6
R1795	R1795C (7 entries)	R1795L (1 entry)				8

References

[1] G. Cardone, J.B. Heymann, A.C. Steven, One number does not fit all: Mapping local variations in resolution in cryo-EM reconstructions, J. Struct. Biol. 184 (2013) 226–236. doi:10.1016/j.jsb.2013.08.002.

[2] Y. Zi Tan, P.R. Baldwin, J.H. Davis, J.R. Williamson, C.S. Potter, B. Carragher, D. Lyumkis, Addressing preferred specimen orientation in single-particle cryo-EM through tilting, Nat. Methods. 14 (2017) 793–796. doi:10.1038/nmeth.4347.

[3] P. Hansmann, A. Brückner, S. Kiontke, B. Berkenfeld, G. Seebohm, P. Brouillard, M. Vikkula, F.E. Jansen, M. Nellist, A. Oeckinghaus, D. Kümmel, Structural analysis of the TSC2 GAP domain: mechanistic insight into catalysis and pathogenic mutations, Structure. (2020) accepted. doi:10.1016/j.str.2020.05.008.

[4] A. Scrima, C. Thomas, D. Deaconescu, A. Wittinghofer, The Rap-{RapGAP} complex: {GTP} hydrolysis without catalytic glutamine and arginine residues., {EMBO} J. 27 (2008) 1145–1153. doi:10.1038/emboj.2008.30.

[5] R. Zech, S. Kiontke, U. Mueller, A. Oeckinghaus, D. Kümmel, Structure of the Tuberous Sclerosis Complex 2 ({TSC2}) N Terminus Provides Insight into Complex Assembly and Tuberous Sclerosis Pathogenesis., J Biol Chem. 291 (2016) 20008–20020. doi:10.1074/jbc.M116.732446.

[6] J. Qin, Z. Wang, M. Hoogeveen-Westerveld, G. Shen, W. Gong, M. Nellist, W. Xu, Structural Basis of the Interaction between Tuberous Sclerosis Complex 1 ({TSC1}) and Tre2-Bub2-Cdc16 Domain Family Member 7 ({TBC1D7})., J Biol Chem. 291 (2016) 8591–8601. doi:10.1074/jbc.M115.701870.

[7] Y. Yu, S. Li, X. Xu, Y. Li, K. Guan, E. Arnold, J. Ding, Structural basis for the unique biological function of small GTPase RHEB, J. Biol. Chem. 280 (2005) 17093–17100. doi:10.1074/jbc.M501253200.
